# Number of Physicians in Chicago

**Published:** 1864-01

**Authors:** 


*Number of Physicians in Chicago.*—We see, by the *Tribune*, that the number of physicians in Chicago, at the close of the year 1863, was three hundred and twenty-two—about one to every five hundred of the inhabitants. This fact may be of interest to our professional brethren who are contemplating to locate among us.

